# Association between iron metabolism and non-alcoholic fatty liver disease: results from the National Health and Nutrition Examination Survey (NHANES 2017–2018) and a controlled animal study

**DOI:** 10.1186/s12986-022-00715-y

**Published:** 2022-12-13

**Authors:** Xinxin Zhang, Ronghua Zuo, Shengjue Xiao, Lirui Wang

**Affiliations:** 1 School of Basic Medicine and Clinical Pharmacy, China Pharmaceutical University, Nanjing, 211198 China; 2 Department of Anesthesiology, The First Affiliated Hospital of Nanjing Medical University, Nanjing, 210029 Jiangsu China; 3 Department of Cardiology, Zhongda Hospital, School of Medicine, Southeast University, Nanjing, 210009 China; 4 Institute of Modern Biology, Nanjing University, 22 Hankou Road, Gulou, Nanjing, 210093 China

**Keywords:** National Health and Nutrition Examination Survey, Non-alcoholic fatty liver disease, Serum iron, Serum ferritin, Transferrin saturation, Soluble transferrin receptor, Hepcidin

## Abstract

**Background:**

Iron metabolism may be involved in the pathogenesis of the non-alcoholic fatty liver disease (NAFLD). The relationship between iron metabolism and NAFLD has not been clearly established. This study aimed to clarify the relationship between biomarkers of iron metabolism and NAFLD.

**Methods:**

Based on the National Health and Nutrition Examination Survey (NHANES), restricted cubic spline models and multivariable logistic regression were used to examine the association between iron metabolism [serum iron (SI), serum ferritin (SF), transferrin saturation (TSAT), and soluble transferrin receptor (sTfR)] and the risk for NAFLD. In addition, stratified subgroup analysis was performed for the association between TSAT and NAFLD. Moreover, serum TSAT levels were determined in male mice with NAFLD. The expression of hepcidin and ferroportin, vital regulators of iron metabolism, were analyzed in the livers of mice by quantitative real-time PCR (qRT-PCR) and patients with NAFLD by microarray collected from the GEO data repository.

**Results:**

Patients with NAFLD showed decreased SI, SF, and TSAT levels and increased STfR levels based on the NHANES. After adjusting for confounding factors, TSAT was significantly negatively correlated with NAFLD. Of note, the relationship between TSAT and NAFLD differed in the four subgroups of age, sex, race, and BMI (*P* for interaction < 0.05). Consistently, mice with NAFLD exhibited decreased serum TSAT levels. Decreased hepcidin and increased ferroportin gene expression were observed in the livers of patients and mice with NAFLD.

**Conclusion:**

Serum TSAT levels and hepatic hepcidin expression were decreased in both patients and mice with NAFLD. Among multiple biomarkers of iron metabolism, lower TSAT levels were significantly associated with a higher risk of NAFLD in the U.S. general population. These findings might provide new ideas for the prediction, diagnosis, and mechanistic exploration of NAFLD.

**Supplementary Information:**

The online version contains supplementary material available at 10.1186/s12986-022-00715-y.

## Introduction

Affecting ~ 25% of the general population worldwide, non-alcoholic fatty liver disease (NAFLD) has become the most common liver disease and is predicted to become increasingly prevalent, particularly among children and younger adults [1, 2]. NAFLD is a progressive disease that can further develop into non-alcoholic steatohepatitis (NASH), liver fibrosis, cirrhosis, and hepatocellular carcinoma, and it is always accompanied by diabetes, cardiovascular disease, and chronic kidney disease, all of which will lead to an increased risk of mortality [3, 4]. The pathogenesis of NAFLD is not fully understood, which is associated with multiplex risk factors [5–7]: metabolic risk factors, gut microbiome composition, genetic factors, epigenetic factors, environmental risk factors, etc.

In recent years, the impact of iron metabolism on NAFLD has attracted renewed attention due to the proposal of “ferroptosis”, an iron-dependent form of cell death [8, 9]. Elena et al. [3] discovered that variants of genes related to iron metabolism are associated with high ferritin levels and increased hepatic iron in an Italian cohort of patients with NAFLD. Jordi et al. [10] showed that iron status influences liver fat accumulation in NAFLD through the gut microbiome. And a rat model of NAFLD exhibited systemic iron deficiency and hepatic iron overload [11]. The above studies support the notion that dysregulated iron homeostasis plays a role in the pathogenesis of NAFLD. And it was reported that a disturbance in iron metabolism affects one-third of patients with NAFLD [12]. However, the association between iron metabolism including serum levels of iron (SI), ferritin (SF), transferrin saturation (TSAT), and soluble transferrin receptor (sTfR) and NAFLD in general population has not been clearly established. Also, whether those biomarkers of iron metabolism could provide certain diagnostic value for NAFLD remains unknown.

Iron, a critical part of the hemoglobin of human beings [12], is essential for oxygen transportation, energy formation, and many cellular functions including DNA synthesis and repair [13, 14]. Iron cannot be naturally created by the human body, but must be ingested through diet or supplements [15]. For men and postmenopausal women, the recommended dietary allowance (RDA) of iron is 8 mg/day, whereas for premenopausal women, it is 18 mg/day according to the Institute of Medicine (US) (Institute of Medicine, 2001). Dietary iron is absorbed mainly in the duodenum and upper jejunum but is not enough for daily needs. The recycled iron from senescent or damaged erythrocytes in the spleen, liver, and bone marrow also contributes to iron storage [16]. Hepcidin is the master regulator for maintaining iron homeostasis, inducing degradation and internalization of the iron exporter ferroportin to inhibit the release of iron from recycling macrophages and absorption from dietary sources, thereby indirectly reducing iron entry into the bloodstream [17, 18]. The liver is the major site of iron storage [19] and plays a central part in iron homeostasis by producing hepcidin [18]. Therefore, liver diseases such as NAFLD may be more susceptible to iron status.

The movement of iron between different cells and body tissues is mainly conducted by the transport protein transferrin, which plays a central role in iron metabolism. Each transferrin protein has two iron-binding domains that can reversibly bind two atoms of iron in a soluble nontoxic form [20, 21]. Serum iron (SI) consists of mostly transferrin-bound iron and a negligible amount of free non-transferrin-bound iron, which is toxic and was reported to promote oxidative stress [22, 23]. Ferritin is a major iron storage protein [24], and serum ferritin (SF) is the small quantity of ferritin circulating in the blood, which is regarded as a marker of iron stores in healthy individuals and those with early iron deficiency (ID). Transferrin saturation (TSAT), which indicates how many transferrin iron-binding sites are occupied, is considered an important biochemical marker of body iron status [25]. sTfR is a soluble form of transferrin receptor identified in serum [26] that reflects the demand for iron in cells and increases rapidly with the depletion of stored iron in the early stage of ID. Four biomarkers, SI, SF, TSAT, and sTfR, are indicators of iron metabolism and are commonly measured in clinics [25, 26].

Although several epidemiological studies have explored associations between biomarkers of iron metabolism and NAFLD, the conclusions were inconclusive and inconsistent. Jung et al. found that serum ferritin levels were positively associated with liver steatosis and fibrosis in the Korean general population [27]. Yang et al. [28] discovered that higher serum iron levels decreased the risk of NAFLD. And to the best of our knowledge, most studies have only evaluated the relationship between one index of iron metabolism and NAFLD. In this study, we comprehensively investigated the association between biomarkers of iron metabolism, including SI, SF, TSAT, and sTfR, and the prevalence of NAFLD in the general population of the U.S., utilizing data from the National Health and Nutrition Examination Survey (NHANES) collected from 2017 to 2018. And we have found that lower TSAT levels were significantly associated with a higher risk of NAFLD, which might provide additional information on biomarkers for the diagnosis of NAFLD. In addition, a controlled animal study was conducted to verify the results of the NHANES.

## Materials and methods

### Population-based human study (NHANES)

#### Study population

The NHAENES database provides a large, sophisticated, stratified, ongoing analysis of nutrition and health data for the entire U.S. population [29]. NHANES data (2017–2018) were used to investigate the relationship between iron metabolism and NAFLD. Those participants who lacked serum iron (SI), serum ferritin (SF), transferrin saturation (TSAT), soluble transferrin (sTfR), and NAFLD data were excluded from this study (n = 918). There was a total of 5483 individuals considered. The National Center for Health Statistics Research Ethics Review Board approved all protocols and informed consent was obtained from all subjects. More information on the survey design, methodology, and data can be found on the NHANES website (https://www.cdc.gov/nchs/nhanes/).

#### NAFLD outcomes

NAFLD was defined using the U.S. fatty liver index (FLI), a well-validated diagnostic index [30], which was employed utilizing NHANES III data and calculated as an equation according to a previous study [31, 32] that included information on body mass index (BMI), gamma glutamyl transferase (GGT), triglycerides (TG), and waist circumference. All the information was collected concurrently with the status of iron metabolism. NAFLD was defined as an FLI score of ≥ 60. The FLI formula is expressed as follows:

$${\text{FLI}} = ({\text{e}}^{{0.953 * \ln ({\text{TG}}) + 0.139 * {\text{BMI}} + 0.718 * \ln ({\text{GGT}}) + 0.053 * {\text{waist circumference}}^{ - 15.745} }} )/(1 + {\text{e}}^{{0.953 * \ln ({\text{TG}}) + 0.139 * {\text{BMI}} + 0.718 * \ln ({\text{GGT}}) + 0.053 * {\text{waist circumference}}^{ - 15.745} }} ) * 100.$$

#### SI, SF, sTfR, and TSAT measurements

The serum iron concentration was measured using the DCX-800 system, which is a timed endpoint method [33]. Acetic acid releases iron from transferrin during the process, and hydroxylamine and thioglycolate reduce it to the ferrous state. FerroZine Iron Reagent quickly complexes with ferrous irons. At a fixed-time interval, the system tracks changes in absorbance at 560 nm. The concentration of iron in the sample is exactly proportional to this change in absorbance. Using the Roche/Hitachi 912 Clinical Analyzer, immunoturbidimetry was the technique of choice for measuring ferritin [34]. Antigen/antibody complexes were created when latex-bound ferritin antibodies interacted with the antigen in the sample. This was turbidimetrically measured after agglutination. The complexes produced were measured at 700 nm and were proportional to the ferritin content (primary wavelength). The method principle for the measurement of soluble transferrin receptor (sTfR) was a particle-enhanced immunoturbidimetric assay that used Roche kits for the Cobas® c501 clinical analyzer [35]. The antigen in the sample reacted with latex particles coated with anti-sTfR antibodies to generate an antigen/antibody combination. The precipitate was identified photometrically after agglutination. Total iron binding capacity (TIBC) was calculated indirectly using the unsaturated iron binding capacity (UIBC) method [36], and the transferrin saturation value was calculated as (iron/TIBC) × 100%. The biomarkers of iron metabolism (SI, SF, TSAT, and sTfR) were divided into quartiles for the assessment of the possible association between iron metabolism and incident NAFLD. The instrument measures SI, SF, TSAT, and sTfR in a range of 11–481 µg/dL, 2–1090 µg/L, 1.4–95% and 1–34.2 mg/L, respectively. Those lower than the detection limits value were missing values and were deleted in this study. The NHANES website (https://wwwn.cdc.gov/nchs/data/nhanes/2017-2018/manuals/2017_MEC_Laboratory_Procedures_Manual.pdf) describes a more thorough processing process for SI, SF, TSAT, and sTfR.

#### Covariates

This research included a number of covariates: demographic data (age, sex, race/ethnicity, family poverty income ratio (PIR), marital status, education level), dietary data (mean energy intake, protein intake, folic acid intake, vitamin B12 intake, vitamin C intake, iron intake), questionnaire data (hypertension, diabetes mellitus (DM), coronary heart disease (CHD), congestive heart failure (CHF), angina pectoris, heart attack, stroke, smoking, alcohol use, and physical activity (PA, which was collected from the Physical Activity questionnaire (PAQ) in NHANES and categorized into four groups according the intensity of PA: No, Moderate, Both (participants who had a combination of moderate-intensity and vigorous-intensity physical activity,), and Vigorous)), examination data (BMI and waist circumference), laboratory data (total cholesterol (TC), triglycerides (TG), high-density lipoprotein cholesterol (HDL cholesterol, HDL-C) and haemoglobin (HB), urinary albumin creatinine ratio (uACR), alanine aminotransferase (ALT), aspartate aminotransferase (AST), GGT, fasting glucose, fasting insulin, glycosylated haemoglobin (HbA1c), high-sensitivity C-reactive protein (hsCRP), estimated glomerular filtration rate (eGFR), uric acid (UA), blood urea nitrogen (BUN), and serum creatinine (Scr)). Detailed covariate information is available publicly from the NHANES database (http://www.cdc.gov/nchs/nhanes/).

#### Statistical analysis

The NHANES estimations were all based on sample weights [37]. All analyses were performed in version 3.6.4 of R (R Foundation for Statistical Computing, Vienna, Austria) and version 22.0 of SPSS (SPSS Inc., Chicago, IL, USA). Continuous variables are presented as the means ± standard deviations, and categorical variables are expressed as numbers (n) and percentages (%). To investigate the relationship between iron metabolism and NAFLD, multivariable logistic regression was used. First, Model 1 was adjusted for age and sex. Second, based on Model 1, race/ethnicity, level of education, marital status, family PIR, hypertension, DM, smoking status, and drinking status were further adjusted for (Model 2). Finally, Model 3 was updated as our main model and included Model 2 variables plus BMI, waist circumference, PA, mean energy intake, protein intake, folic acid intake, vitamin B12 intake, vitamin C intake, iron intake, the complication of CHD, CHF, angina pectoris, heart attack, stroke, and TC, TG, HDL-C, HB, HbA1c, hsCRP, ALT, AST, GGT, eGFR, uACR, UA, BUN, and Scr. Subgroup analyses were used to evaluate the relationship between iron status and NAFLD based on age, sex, race/ethnicity, hypertension, DM, and BMI. We used regularization technique (Least absolute shrinkage and selection operator (LASSO) regression) to solve the potential overfitting problem. In addition, multivariate stepwise regression analysis was performed to deal with multiple testing problem. *P-*value < 0.05 was considered statistically significant.

### Laboratory-based controlled animal study

Five-week-old male C57BL/6J mice were purchased from Beijing Vital River Laboratory Animal Technology Co., Ltd., treated according to the guidelines of the China Pharmaceutical University Institutional Animal Care and Use Committee (approval number: 2019-03-001), and all mouse studies were reported according to the ARRIVE guidelines [38]. Mice were housed in a specific pathogen-free (SPF) facility and adapted to the new conditions for 2 days before the experiment. Mice were maintained in a temperature-controlled (22–23 °C) room with a 12:12-h light/dark cycle.

#### Animals and experimental protocols

To induce NAFLD, mice were fed a high-fat diet (D12492, 60% kcal from fat; research diet) and fructose (Y0002132, 2.31 g/100 ml; sigma) water (high-fat-fructose diet) for 30 weeks (n = 5). Mice fed normal chow served as controls (n = 5). When the mice were sacrificed, blood was collected from the inferior vena cava, and the entire middle lobe of the liver was fixed with formalin. The largest lobe in the middle of the liver was divided into 8 small pieces after cutting the edge, and one of those 8 pieces was directly added to TRIzol (9109, TaKaRa, Dalian, Liaoning, China) for RNA extraction.

#### Assays for biomarkers of iron metabolism

Transferrin saturation (TSAT) was defined as the ratio of serum iron (SI) and total iron-binding capacity (TIBC). Unsaturated iron-binding capacity (UIBC) is calculated by subtracting SI from TIBC [39]. SI and TIBC were measured using commercial kits (all from Nanjing Jiancheng Bioengineering Institute) according to standard procedures [11, 40]. Serum ferritin, sTfR, and transferrin were determined using ELISA kits (Jiangsu Meibiao Biotechnology Co., Ltd) according to the manufacturer’s instruction [41]. Serum ferrous irons (Fe^2+^) serum was measured using the phenanthroline colorimetric method [40] by colorimetric assay kit purchased from Elabscience following the manufacturer’s instructions.

#### Measurements of hepatic TG, TC and MDA

Three pieces of liver were selected for the quantification of triglyceride (TG), total cholesterol (TC), and malonic dialdehyde (MDA) respectively, which were determined by commercial kits (Nanjing Jiancheng Bioengineering Inst ferrous iron itute) according to the manufacturer’s protocols as previously described [5, 42]. Thiobarbituric acid (TBA) method was used for the determination of MDA, which was based on that MDA can react with TBA at high temperature and acidity to produce the red-brown product with the maximum absorption peak at 532 nm [42].

#### Histological analysis

The fixed liver lobes were embedded in paraffin, then sectioned at 5 µm and stained with hematoxylin–eosin (H&E) according to the standard protocol described [43–45]. Scores for steatosis, inflammation, and ballooning were assessed by a four-member research team according to the NAFLD Activity Score (NAS) system [46].

#### RNA extraction and quantitative real-time PCR (qRT‒PCR) analysis

Total RNA extraction and cDNA reversion were conducted according to methods described previously [5, 7, 47]. qRT-PCR was performed using SYBR Premix (Vazyme, Q331-02, China) according to the manufacturer’s instructions for the ABI StepOnePlus real-time PCR machine (Applied Biosystems). Custom primers were designed for mouse hepcidin (forward, 5′-TTGCGATACCAATGCAGAAGA-3′; reverse, 5′-GATGTGGCTCTAGGCTATGTTTTG-3′), ferroportin (forward, 5′-TTGCAGGAGTCATTGCTGCTA-3′; reverse: 5′-TGGAGTTCTGCACACCATT-3′), and 18S (forward, 5′-AGTCCCTGCCCTTTGTACACA-3′; reverse, 5′-CGATCCCAGGGCCTCACTA-3′). All real-time PCR primers were synthesized by Sangon Biotech (Shanghai, China). Gene expression was normalized to 18S and calculated as previously described [5, 7, 47].

#### Statistical analysis

The results are presented as the means ± SEMs and were statistically analysed by the unpaired Student's t test or Pearson product-moment correlation coefficients. Differences were considered significant when the *P*-value was < 0.05.

### Microarray and RNA-seq data

Hepatic gene expression was compared among 20 patients with simple steatosis, 19 with non-alcoholic steatohepatitis (NASH), and 24 healthy controls in the GSE89632 dataset [48] (https://www.ncbi.nlm.nih.gov/geo/query/acc.cgi?acc=GSE89632). In the GSE126848 dataset, RNA sequencing was performed on liver biopsies obtained from healthy normal weight (n = 14) and healthy obese (n = 12) individuals, and simple steatosis (n = 15) and NASH (n = 16) patients [49] (https://www.ncbi.nlm.nih.gov/geo/query/acc.cgi?acc=GSE126848). In the GSE185051 dataset, liver biopsies from 51 paediatric NASH patients and 5 normal subjects were analysed by RNA sequencing [50] (https://www.ncbi.nlm.nih.gov/geo/query/acc.cgi?acc=GSE185051). 12 biopsy diagnosed NASH patients and 5 different subjects for healthy control groups were included in the GSE24807 dataset [51] (https://www.ncbi.nlm.nih.gov/geo/query/acc.cgi?acc=GSE24807).

To obtain these data, we used the package 'GEOquery' installed in R, especially the function getGEO(),  importing the dataset GSE number, and obtained the list object of the corresponding dataset. Then, the pData() and expr() functions were used to view the sample information and expression matrix, respectively. The chip platform used in the dataset was confirmed by viewing the web page and checking the list of objects. The gene annotation information of the platform was obtained by using the GEOquery software package and matched with the array information. The gene symbol was used to replace the probe name. Genes with |log2-fold changes (FCs)|> 0.5 and *P*-value < 0.05 were considered differentially expressed genes (DEGs). Then, the hepcidin and ferroportin genes were selected, and the expression information of these genes in each sample and the grouping information of each sample were extracted and stored in an Excel table for subsequent analysis. The differences between different groups were compared by the Mann–Whitney test and considered statistically significant at the *P-*value < 0.05.

## Results

### Population-based human study (NHANES)

#### Baseline characteristics

The population-weighted characteristics of this study are presented in Table 1. The final analysis consisted of 5483 individuals after the removal of people with missing iron metabolism index (SI, SF, TSAT, and sTfR) and NAFLD information. The average age of the entire population was 44.425 ± 0.622 years. The prevalence of NAFLD was 35.19% in this group. In addition, the SI, SF, TSAT, and sTfR averages among these 5483 individuals were 89.073 ± 1.107 µg/dL, 138.120 ± 3.320 µg/L, 27.489 ± 0.291%, and 3.211 ± 0.047 mg/L, respectively. Compared with participants without NAFLD, participants with NAFLD showed significantly decreased levels of SI (*P* < 0.001) and TSAT (*P* < 0.001) and a significantly increased level of sTfR (*P* < 0.001) (Table 1). However, SF levels did not differ significantly between the two groups. (*P* = 0.342), even though it was slightly decreased in NAFLD groups on average. Among 918 participants with missing data, 12.5% were Mexcan American, 9.6% were Other Hispanic, 25.2% were Non-Hispanic Black, 32.4% were Non-Hispanic White, and 20.4% were Other race. Those under the age of 60 accounted for 67.7%, and individuals 60 years of age or older accounted for 32.3%. In addition, those were male accounted for 46.1%, and individuals were female accounted for 53.9%. Finally, we also compared the characteristics of the populations between the those with missing values and those without in Additional file 5: Table S1.

**Table 1:** Characteristics of the study population

Variable	Overall (n = 5483)	Non-NAFLD (n = 3553)	NAFLD (n = 1930)	*P*-value
Age, years	44.425 ± 0.622	41.660 ± 0.551	50.490 ± 0.723	< 0.001
Sex, %				0.006
Male	2705 (49.3%)	1851 (52.1%)	854 (44.2%)	
Female	2778 (50.7%)	1702 (47.9%)	1076 (55.8%)	
Race, %				0.237
Mexican American	810 (14.8%)	497 (14.0%)	313 (16.2%)	
Other hispanic	511 (9.3%)	329 (9.3%)	182 (9.4%)	
Non-hispanic black	1216 (22.2%)	801 (22.5%)	415 (21.5%)	
Non-hispanic white	1868 (34.1%)	1218 (34.3%)	650 (33.7%)	
Other race	1078 (19.7%)	708 (19.9%)	370 (19.2%)	
Family PIR	2.999 ± 0.062	3.032 ± 0.060	2.926 ± 0.083	0.102
Education level, %				0.691
Less than high school	1632 (29.8%)	1046 (29.4%)	586 (30.4%)	
High school	1212 (22.1%)	787 (22.2%)	425 (22.0%)	
More than high school	2639 (48.1%)	1720 (48.4%)	919 (47.6%)	
Marital status, %				< 0.001
Having a partner	3031 (55.3%)	1912 (53.8%)	1119 (58.0%)	
No partner	1054 (19.2%)	614 (17.3%)	440 (22.8%)	
Unmarried	1398 (25.5%)	1027 (28.9%)	371 (19.2%)	
Hypertension, %				< 0.001
No	3342 (61.0%)	2361 (66.5%)	978 (50.7%)	
Yes	2141 (39.0%)	1189 (33.5%)	952 (49.3%)	
DM, %				< 0.001
No	4491 (81.9%)	3097 (87.2%)	1394 (72.2%)	
Yes	992 (12.8%)	456 (12.8%)	536 (27.8%)	
Smoker, %				0.083
No	3328 (60.7%)	2141 (60.3%)	1187 (61.5%)	
Former	1157 (21.1%)	697 (19.6%)	460 (23.8%)	
Now	998 (18.2%)	715 (20.1%)	283 (14.7%)	
Alcohol user, %				< 0.001
No	873 (15.9%)	354 (10.0%)	519 (26.9%)	
Mild	2233 (40.7%)	1509 (42.5%)	724 (37.5%)	
Moderate	1045 (19.1%)	741 (20.9%)	304 (15.8%)	
Heavy	1332 (24.3%)	949 (26.7%)	383 (19.8%)	
CHD, %				< 0.001
No	5291 (96.5%)	3457 (97.3%)	1834 (95.0%)	
Yes	192 (3.5%)	96 (2.7%)	96 (5.0%)	
CHF, %				0.007
No	5360 (97.8%)	3493 (98.3%)	1867 (96.7%)	
Yes	123 (2.2%)	60 (1.7%)	63 (3.3%)	
Angina pectoris, %				0.023
No	5356 (97.7%)	3488 (98.2%)	1868 (96.8%)	
Yes	127 (2.3%)	65 (1.8%)	62 (3.2%)	
Heart attack, %				0.033
No	5278 (96.3%)	3439 (96.8%)	1839 (95.3%)	
Yes	205 (3.7%)	114 (3.2%)	91 (4.7%)	
Stroke, %				0.017
No	5274 (96.2%)	3434 (96.7%)	1840 (95.3%)	
Yes	209 (3.8%)	119 (3.3%)	90 (4.7%)	
PA, %				0.015
No	2855 (52.1%)	1790 (50.4%)	1065 (55.2%)	
Moderate	1211 (22.1%)	785 (22.1%)	426 (22.1%)	
Both	1164 (21.2%)	800 (22.5%)	364 (18.9%)	
Vigorous	253 (4.6%)	178 (5.0%)	75 (3.9%)	
Mean energy	2074.407 ± 21.096	2106.648 ± 28.210	2003.693 ± 22.084	0.011
Intake (kcal/day)				
Protein intake, g	79.987 ± 1.152	81.589 ± 1.519	76.474 ± 1.306	0.020
Folic acid intake, mcg	175.012 ± 3.764	180.048 ± 4.461	163.965 ± 4.359	0.008
Vitamin B12 intake, mcg	4.757 ± 0.084	4.880 ± 0.116	4.487 ± 0.099	0.028
Vitamin C intake, mg	73.632 ± 1.368	75.895 ± 1.777	68.670 ± 1.151	0.002
Iron intake, mg	14.124 ± 0.196	14.347 ± 0.260	13.635 ± 0.212	0.046
BMI, kg/m^2^	29.138 ± 0.243	27.900 ± 0.246	31.854 ± 0.349	< 0.001
Waist circumference, cm	98.776 ± 0.636	95.438 ± 0.681	106.097 ± 0.798	< 0.001
Hb, g/dL	14.207 ± 0.059	14.304 ± 0.061	13.994 ± 0.073	< 0.001
Hs-CRP, mg/L	3.588 ± 0.145	2.990 ± 0.137	4.899 ± 0.332	< 0.001
HbA1c, %	5.629 ± 0.015	5.523 ± 0.017	5.861 ± 0.026	< 0.001
ALT, U/L	22.515 ± 0.378	24.199 ± 0.474	18.823 ± 0.440	< 0.001
AST, U/L	22.094 ± 0.264	23.372 ± 0.384	19.290 ± 0.305	< 0.001
GGT, U/L	28.258 ± 0.502	29.288 ± 0.700	25.999 ± 0.797	0.013
TC, mg/dL	185.497 ± 1.588	184.524 ± 1.722	187.630 ± 1.521	0.015
TG, mg/dL	136.928 ± 3.177	129.096 ± 4.015	154.106 ± 2.392	< 0.001
HDL-C, mmol/L	1.381 ± 0.012	1.414 ± 0.014	1.309 ± 0.011	< 0.001
BUN, mg/dL	14.589 ± 0.168	14.444 ± 0.175	14.909 ± 0.180	0.003
UA, mg/dL	5.344 ± 0.035	5.284 ± 0.048	5.475 ± 0.036	0.008
Scr, mg/dL	0.862 ± 0.006	0.858 ± 0.007	0.871 ± 0.008	0.147
eGFR, ml/min/1.73m^2^	98.810 ± 0.853	101.082 ± 0.806	93.826 ± 0.969	< 0.001
uACR	34.535 ± 3.264	31.025 ± 3.231	42.232 ± 5.562	0.053
SI, µg/dL	89.073 ± 1.107	92.688 ± 1.348	81.144 ± 1.441	< 0.001
SF, µg/L	138.120 ± 3.320	140.087 ± 4.352	133.804 ± 4.726	0.342
TSAT, %	27.489 ± 0.291	28.671 ± 0.397	24.897 ± 0.378	< 0.001
sTfR, mg/L	3.211 ± 0.047	3.114 ± 0.049	3.423 ± 0.070	< 0.001

NAFLD, Non-alcoholic fatty liver disease; family PIR, family poverty income ratio; DM, diabetes mellitus; BMI, body mass index, PA, physical activity; Hb, hemoglobin; hs-CRP, high-sensitivity C-reactive protein; HbA1c, glycosylated hemoglobin; ALT, alanine aminotransferase; AST, aspartate aminotransferase; GGT, gamma-glutamyl transpeptidase; TC, total cholesterol; TG, triglycerides; HDL-C, high- density lipoprotein-cholesterol, BUN, blood urea nitrogen; UA, uric acid; Scr, serum creatinine; eGFR, estimated glomerular filtration rate; uACR, urinary albumin creatinine ratio; SI, serum iron; SF, serum ferritin; TSAT, Transferrin saturation; sTfR, soluble transferrin receptor

In healthy individuals, the normal ranges SI, SF, and TSAT in adults are 55–185 µg/dL, 15–250 µg/L and 14.4–46.8% respectively [52, 53]. As for the normal range of sTfR, different measurement methods provide different values due to the lack of uniform standards [26, 54]. The iron status index was calculated and divided into quartiles in this study. The SF ranged from < 42.50, 42.50 to 93.30, 93.31 to 179.00, and > 179.00 in quartile 1 (Q1), quartile 2 (Q2), quartile 3 (Q3), and quartile 4 (Q4), respectively; the SI ranged from < 63.00, 63.00 to 84.00, 84.01 to 108.00, and > 108.00 in quartile 1 (Q1), quartile 2 (Q2), quartile 3 (Q3), and quartile 4 (Q4), respectively; the TSAT ranged from < 19.00, 19.00 to 26.00, 26.01 to 34.00, and > 34.00 in quartile 1 (Q1), quartile 2 (Q2), quartile 3 (Q3), and quartile 4 (Q4), respectively; and the sTfR ranged from < 2.49, 2.49 to 2.99, 3.00 to 3.68, and > 3.68 in quartile 1 (Q1), quartile 2 (Q2), quartile 3 (Q3), and quartile 4 (Q4), respectively (Table 2).

**Table 2:** Adjusted ORs for associations between iron metabolism and the prevalence of NAFLD

Iron metabolism	Model 1		Model 2		Model 3	
	OR (95%CI)	*P* for trend	OR (95%CI)	*P* for trend	OR (95%CI)	*P* for trend
SI (µg/dl)		< 0.001		< 0.001		0.213
Q1(< 63.00)	1.00		1.00		1.00	
Q2 (63.00–84.00)	0.676 (0.450, 0.915)*		0.836 (0.713, 0.981)*		1.007 (0.844, 1.203)	
Q3 (84.01–108.00)	0.621 (0.505, 0.765)***		0.762 (0.647, 0.897)***		0.994 (0.825, 1.197)	
Q4 (> 108.00)	0.378 (0.272, 0.526)***		0.532 (0.448, 0.632)***		0.842 (0.688, 1.030)	
SF (µg/l)		0.239		0.530		0.616
Q1 (< 42.50)	1.00		1.00		1.00	
Q2 (42.50–93.30)	0.973 (0.823, 1.149)		1.022 (0.862, 1.211)		1.093 (0.905, 1.320)	
Q3 (93.31–179.00)	0.916 (0.769, 1.092)		1.000 (0.836, 1.196)		1.026 (0.837, 1.258)	
Q4 (> 179.00)	0.838 (0.696, 1.008)		0.905 (0.749, 1.095)		0.973 (0.779, 1.214)	
TSAT (%)		< 0.001		< 0.001		0.002
Q1 (< 19.00)	1.00		1.00		1.00	
Q2 (19.00–26.00)	0.751 (0.642, 0.878)***		0.789 (0.672, 0.925)**		0.905 (0.758, 1.079)	
Q3 (26.01–34.00)	0.715 (0.609, 0.839)***		0.785 (0.666, 0.925)**		0.987 (0.821, 1.186)	
Q4 (> 34.00)	0.433 (0.363, 0.516) ***		0.480 (0.401, 0.575) ***		0.706 (0.575, 0.868) **	
sTfR (mg/L)		< 0.001		< 0.001		0.453
Q1 (< 2.49)	1.00		1.00		1.00	
Q2 (2.49–2.99)	1.116 (0.989, 1.376)		1.121 (0.947, 1.328)		1.031 (0.961, 1.235)	
Q3 (3.00–3.68)	1.406 (1.191, 1.652)***		1.291 (1.092, 1.526)**		1.096 (0.916, 1.312)	
Q4 (> 3.68)	1.736 (1.476, 2.042)***		1.523 (1.289, 1.800)***		1.151 (0.955, 1.387)	

SI, Serum iron; SF, serum ferritin; TSAT, Transferrin saturation; sTfR, soluble transferrin receptor; NAFLD, non-alcoholic fatty liver disease; Model 1: age and sex. Model 2: Model 1 variables plus race/ethnicity, family poverty-income ratio, marital status, education level, hypertension, diabetes mellitus, smoker, alcohol user. Model 3 was adjusted for Model 2 variables plus body mass index, waist circumference, physical activity, the complication of CHD, CHF, angina pectoris, heart attack, and stroke, mean energy intake, protein intake, folic acid intake, Vitamin B12 intake, Vitamin C intake, Iron intake, high-sensitivity C-reactive protein, glycosylated hemoglobin, alanine aminotransferase, aspartate aminotransferase, gamma-glutamyl transpeptidase, blood urea nitrogen, uric acid, serum creatinine, estimated glomerular filtration rate, urinary albumin creatinine ratio, hemoglobin, high-density lipoprotein-cholesterol, total cholesterol, triglycerides; *P < 0.05, **P < 0.01, ***P < 0.001: All *P*-values were calculated using Q1 as the reference. *P* for the trend is presented as the differences between Q1, Q2, Q3 and Q4

### Association between iron status and NAFLD

**Fig. 1:**
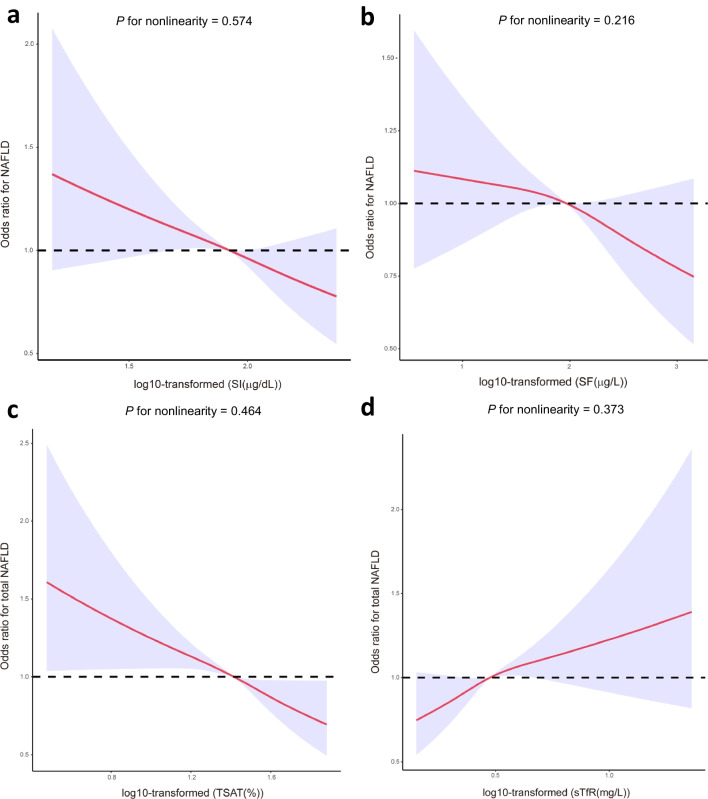
Restricted cubic spline plots of the association between the indicators of iron metabolism and the risk of NAFLD based on the NHANES database. The levels of serum iron (**a**), ferritin (**b**), TSAT (**c**), and sTfR (**d**) were log10 transformed. Analyses were adjusted for age, sex, education level, race/ethnicity, family poverty-income ratio, smoking, hypertension, diabetes mellitus, body mass index, waist circumference, physical activity, total energy intake, high-density lipoprotein-cholesterol, triglycerides, total cholesterol, uric acid, and hemoglobin. Solid and dashed lines represent the log-transformed odds ratios and the corresponding 95% confidence intervals

The potential relationship between each iron status index and NAFLD risk was examined using restricted cubic splines with a three-knot model. There was a linear and negative correlation between SI, SF, as well as TSAT and NAFLD (Fig. 1a–c), whereas there was a linear positive correlation between sTfR and NAFLD (Fig. 1d). In addition, the correlation heatmap of biomarkers of iron metabolism and NAFLD revealed that SI was negatively related with NAFLD (r =  − 0.13), SF was negatively related with NAFLD (r =  − 0.01), TSAT was negatively related with NAFLD (r =  − 0.13), and sTfR were positively related with NAFLD (r = 0.06) (Additional file 1: Fig. S1). Table 2 illustrates the results of the multivariate logistic regression analyses for the iron metabolism index (SI, SF, TSAT, and sTfR) and NAFLD. After adjustment for confounding factors, compared with people in the Q1 group, the odds ratios (ORs) with 95% confidence intervals (CIS) for NAFLD were 1.007 (0.844, 1.203), 0.994 (0.825, 1.197), and 0.842 (0.688, 1.030) for SI; 1.093 (0.905, 1.320), 1.026 (0.837, 1.258), and 0.973 (0.779, 1.214) for SF; 0.905 (0.758, 1.079), 0.987 (0.821, 1.186), and 0.706 (0.575, 0.868) for TSAT; and 1.031 (0.961, 1.235), 1.096 (0.916, 1.312), and 1.151 (0.955, 1.387) for sTfR in the Q2, Q3 and Q4 groups, respectively. In multivariate logistic regression model, TSAT levels were significantly inversely correlated with NAFLD. In addition, iron metabolism markers (SI, SF, TSAT, and sTfR) were used as continuous variables to explore their association with NAFLD (Additional file 6: Table S2). A total of 43 features were reduced to 13 potential predictors, including TSAT, on the basis of 5483 individuals, and were features with nonzero coefficients in the LASSO regression model (Additional file 2: Fig. S2a, b). We used the other 12 features (age, BMI, waist, Alt, Ast, HDL-C, HbA1c, protein intake, sex, education level, DM, and alcohol user) as covariates screened by LASSO regression analysis to explore the association between TSAT and NAFLD. The RCS plot also showed a liner and negative relationship between TSAT and NAFLD (Additional file 3: Fig. S3a). Additionally, by using multivariate stepwise regression analysis, we reduced 43 features to 13 potential predictors, including TSAT (Additional file 7: Table S3). Then, we used the other 12 covariates (age, BUN, smoker, waist, Alt, HbA1C, sex, TC, alcohol user, HDL-C, education level, and dietary Vitamin C intake) as covariates screened by multivariate stepwise regression analysis to explore the relationship between TATS and NAFLD. The RCS plot also showed a liner and negative relationship between TSAT and NAFLD (Additional file 3: Fig. S3b).

#### Subgroup analysis

Subgroup analyses stratified by age, sex, race, hypertension, DM, and BMI were further conducted (Table 3). As shown in Table 3, the association between TSAT and NAFLD was statistically significant in those subgroups of individuals aged < 60 years (*P* for trend = 0.009), female (*P* for trend = 0.019), Mexican American (*P* for trend = 0.018), participants without hypertension (*P* for trend = 0.003) or DM (*P* for trend = 0.013), and individuals with a BMI of ≥ 30 kg/m^2^ (*P* for trend = 0.033). Notably, the association differed in the four subgroups of age, sex, race, and BMI (*P* for interaction < 0.05).

**Table 3:** Subgroups analysis for the associations of TSAT with the prevalence of NAFLD

TSAT (%)	Q1 (< 19.00)	Q2 (29.00–26.00)	Q3 (26.01, 34.00)	Q4 (> 34.00)	*P* for trend	*P* for interaction
	OR (95%CI)	OR (95%CI)	OR (95%CI)	OR (95%CI)		
Age						0.021
< 60	1.00 (Ref.)	0.892 (0.719, 1.108)	0.973 (0.774, 1.221)	0.667 (0.516, 0.863)**	0.009	
≥ 60	1.00 (Ref.)	1.021 (0.745, 1.400)	1.189 (0.859, 1.644)	0.831 (0.579, 1.193)	0.154	
Sex						0.002
Male	1.00 (Ref.)	0.846 (0.638, 1.122)	0.830 (0.622, 1.107)	0.644 (0.492, 0.896)**	0.050	
Female	1.00 (Ref.)	0.940 (0.743, 1.188)	1.136 (0.886, 1.459)	0.697 (0.513, 0.948)*	0.019	
Race						0.020
Mexican American	1.00 (Ref.)	0.655 (0.410, 1.046)	0.816 (0.497, 1.341)	0.427 (0.244, 0.748)**	0.018	
Other hispanic	1.00 (Ref.)	0.931 (0.487, 1.780)	1.491 (0.776, 2.862)	0.841 (0.429, 1.645)	0.291	
Non-hispanic white	1.00 (Ref.)	0.959 (0.660, 1.391)	1.044 (0.698, 1.562)	0.978 (0.615, 1.555)	0.975	
Non-hispanic black	1.00 (Ref.)	0.844 (0.641, 1.220)	0.963 (0.693, 1.338)	0.669 (0.461, 0.971)*	0.122	
Other race	1.00 (Ref.)	1.064 (0.701, 1.614)	0.921 (0.602, 1.407)	0.648 (0.402, 1.046)	0.150	
Hypertension						0.556
No	1.00 (Ref.)	0.882 (0.694, 1.119)	0.959 (0.747, 1.230)	0.622 (0.470, 0.822)**	0.003	
Yes	1.00 (Ref.)	0.970 (0.741, 1.269)	1.079 (0.816, 1.427)	0.841 (0.612, 1.154)	0.398	
DM						0.598
No	1.00 (Ref.)	0.917 (0.750, 1.122)	1.053 (0.856, 1.296)	0.747 (0.592, 0.943)*	0.013	
Yes	1.00 (Ref.)	0.869 (0.593, 1.272)	0.807 (0.534, 1.220)	0.622 (0.386, 1.002)	0.269	
BMI						0.001
< 30	1.00 (Ref.)	0.897 (0.696, 1.156)	0.901 (0.697, 1.165)	0.707 (0.534, 0.936)	0.084	
≥ 30	1.00 (Ref.)	0.918 (0.712, 1.183)	1.158 (0.875, 1.531)	0.725 (0.524, 1.004)	0.033	

TSAT, Transferrin saturation; NAFLD, non-alcoholic fatty liver disease; DM, diabetes mellitus; BMI, body mass index; Analysis was adjusted for age, sex, race/ethnicity, education level, marital status, family poverty income ratio, hypertension, diabetes mellitus, smoker, alcohol user, body mass index, waist circumference, physical activity, the complication of CHD, CHF, angina pectoris, heart attack, and stroke, mean energy intake, protein intake, folic acid intake, Vitamin B12 intake, Vitamin C intake, Iron intake, high-sensitivity C-reactive protein, glycosylated hemoglobin, alanine aminotransferase, aspartate aminotransferase, gamma-glutamyl transpeptidase, blood urea nitrogen, uric acid, serum creatinine, estimated glomerular filtration rate, urinary albumin creatinine ratio, hemoglobin, high-density lipoprotein-cholesterol, total cholesterol, triglycerides

### Laboratory-based controlled animal study

**Fig. 2:**
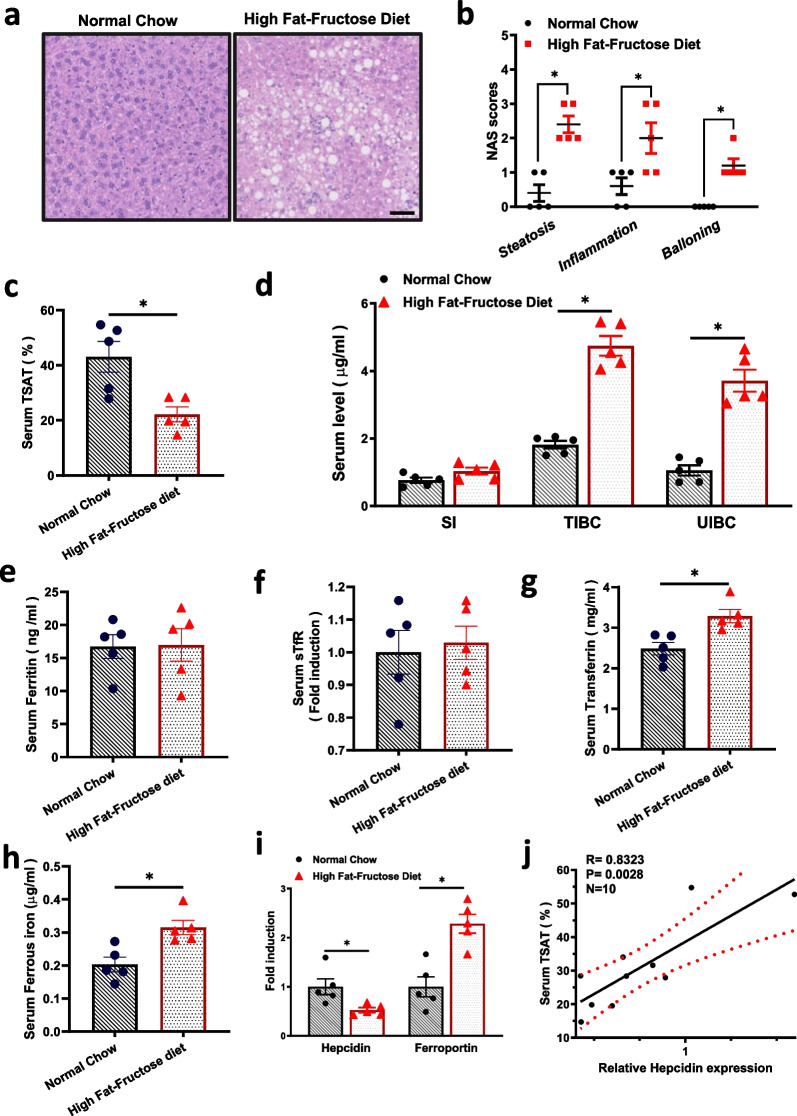
Decreased serum TSAT and hepatic hepcidin expression were shown in mice with NAFLD. **a**–**j** Mice were fed a normal chow or high-fat-fructose diet for 30 weeks (n = 5 for each group). **a** Representative H&E staining of the liver sections. Scale bars: 50 µm. **b** NAS scores for quantifying H&E staining. **c** Levels of serum TSAT. **d** Levels of serum SI, TIBC and UIBC. **e** Levels of serum ferritin. **f** Levels of serum sTfR. **g** Levels of serum transferrin. **h** Levels of serum ferrous iron (Fe^2+^). **i** Relative mRNA levels of hepcidin and ferroportin in the livers of the mice in the indicated groups. **j** Correlation of liver hepcidin expression with serum TSAT levels

Since we found a significant inverse relationship between TSAT and NAFLD based on the NHANES survey, we constructed a high-fat-fructose diet NAFLD model in an animal study, which most closely resembled human NAFLD [55]. According to the statistical information in the review article [55], we selected male mice for the experiment, excluding the unstable factors that may be caused by estrogen or progesterone in female mice. Compared with mice fed a normal chow diet, mice fed a high-fat-fructose diet for 30 weeks showed significant weight gain, as well as significant increases in TG and TC levels in the liver (Additional file 4: Fig. S4a,b). Biopsy of liver tissue is the gold standard for the diagnosis of NAFLD [56]. As shown in Fig. 2a, liver sections from mice fed a high-fat-fructose diet exhibited more substantial liver fat accumulation, inflammatory cell infiltration, and hepatocyte distension, as quantified by NAS scores (Fig. 2b), compared with mice fed a normal chow diet. These results demonstrated the successful establishment of the NAFLD model in mice. We then determined the level of serum TSAT and found that it was significantly decreased in mice with NAFLD (Fig. 2c), which was consistent with the NHANES database. However, SI levels did not show the same downwards trend among mice with NAFLD as in the NHANES database. For the assessment of iron status in human, SI was not very informative because SI values vary greatly and even fluctuate hourly [11]. Both TIBC and UIBC were significantly elevated, as shown in Fig. 2d, which associated with a decrease in TSAT levels (as TSAT was defined as the ratio of SI in TIBC). Next, we evaluated serum ferritin and sTfR in the mouse model by ELISA and found that neither of them had significant changes (Fig. 2e–f). However, serum transferrin was significantly increased, which was consistent with the increase in TIBC (Fig. 2g). In addition, we measured serum ferrous irons (Fe^2+^) and found it was increased significantly in mice with NAFLD (Fig. 2h**)**. Fe^2+^ is the key to producing the Fenton reaction [57], which might be related to excessive oxidative stress and liver injure proved by increased hepatic MDA levels in NAFLD (Additional file 4: Fig. S4c).

Hepcidin is a major regulator of iron metabolism, mainly by regulating the degradation of the iron exporter ferroportin [17, 18]. Under iron deficiency, the expression of hepcidin is reduced to ensure that more iron enters the blood and maintains iron balance [58]. We evaluated the mRNA expression of hepcidin in the liver and found that it was significantly decreased, while that of ferroportin was increased (Fig. 2i). The correlation between TSAT and hepcidin is not very clear [59, 60]; we examined this association and found that TSAT and hepcidin were positively correlated (Fig. 2j).

### Microarray and RNA-seq data

**Fig. 3:**
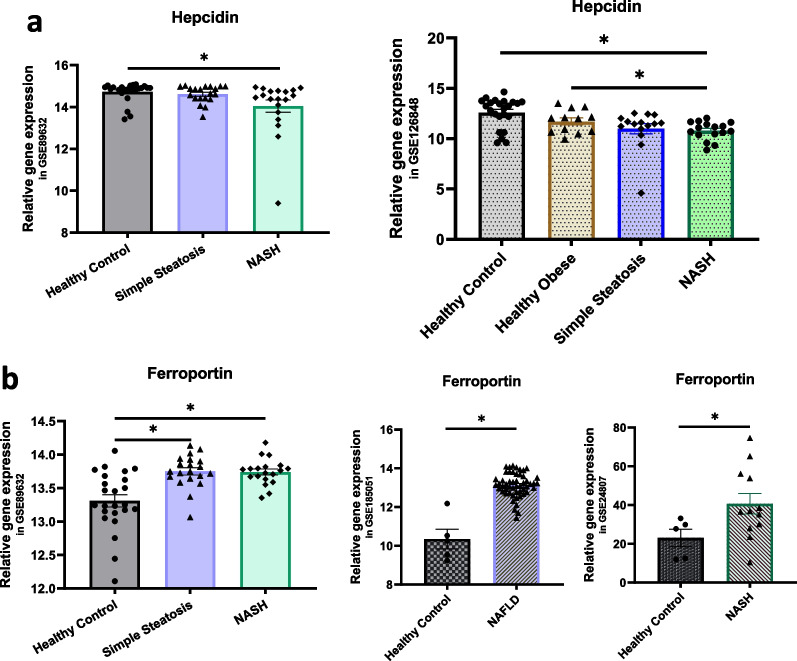
Human hepcidin and ferroportin gene expression acquired from the Gene Expression Omnibus datasets. **a** Human hepcidin expression in the GSE89632 and GSE126848 databases. **b** Human ferroportin expression in the GSE89632, GSE185051 and GSE24807 databases

Next, we investigated the transcriptional levels of hepcidin and ferroportin in the livers of NAFLD patients using Gene Expression Omnibus (GEO) datasets. Compared with healthy individuals, patients with NAFLD had decreased hepatic hepcidin (Fig. 3a) and increased hepatic ferroportin (Fig. 3b) gene expression [48–51].

In summary, the current study comprehensively investigated the association between biomarkers of iron metabolism, including SI, SF, TSAT, and sTfR, and the prevalence of NAFLD in the general U.S. population. We found that decreased levels of SI, SF, and TSAT were associated with a high risk of NAFLD, while increased sTfR levels were associated with an elevated prevalence of NAFLD. We then used multivariate logistic regression analyses to assess the association between these four indicators and NAFLD and found that only the correlation between TSAT and NAFLD was statistically significant. The association was statistically significant among individuals younger than 60 years, woman, Mexican American, and those who had a BMI ≥ 30 kg/m^2^ (*P* for trend < 0.05). In addition, a controlled animal study was conducted to verify the results of the NHANES. Compared with mice fed normal chow, mice with NAFLD fed a high-fat-fructose diet showed decreased serum TSAT levels. Moreover, we analyzed the expression of hepcidin and ferroportin, vital regulators of iron metabolism, and observed decreased hepcidin and increased ferroportin gene expression in the livers of patients and mice with NAFLD. We clearly elucidated the relationship between iron metabolism indicators and NAFLD.

## Discussion

The current study investigated the association between iron metabolism and the prevalence of NAFLD in a large-scale American population and a controlled animal study. Based on the NHANES database, decreased serum TSAT levels were found in patients with NAFLD and were significantly correlated with a higher risk of NAFLD. A subgroup analysis for the correlation between TSAT and NAFLD stratified by age, sex, hypertension, DM, and BMI was further performed and exhibited different associations in the four subgroups. Consistent with the results from the NHANES, mice with NAFLD also exhibited decreased serum TSAT levels, which was correlated with reduced liver hepcidin gene expression. Additionally, microarray data showed that hepcidin is decreased at the transcriptional level in NAFLD patients. These findings indicate the dysregulation of iron metabolism in NAFLD and might provide additional information on biomarkers for the diagnosis of NAFLD in the general population.

Iron, as an essential trace element in human beings, is abundant on earth. However, the bioavailability of iron is hampered by its ability to form highly insoluble oxides, with iron deficiency being the most common nutritional disorder, affecting billions of people worldwide [61]. In contrast to the high prevalence of iron deficiency, iron overload is less prevalent but is often observed among patients with NAFLD [62]. Excess iron is toxic, as it exacerbates oxidative stress and severely damages cells and tissues via the Fenton reaction [63]. Among the four indicators (SI, SF, TSAT and sTfR) of iron metabolism, TSAT was significantly decreased both in mice and patients with NAFLD. The mRNA level of the hepatic hepcidin, key regulator of iron metabolism, was also significantly decreased in mice and patients with NAFLD. The changes in TSAT and hepcidin indicated the dysregulation of iron metabolism in NAFLD. Among NAFLD patients of the NHANES, the average value of TSAT was 24.897 ± 0.378, which was still within the normal range. A decrease in TSAT levels indicated a reduction in the proportion of transferrin-bound iron. Transferrin is a class of glycoproteins that are easily modified by glycosylation [64]. Against the background of NAFLD, some transferrin may be abnormally glycosylated [65] and cannot bind and transport iron, resulting in an increase in unavailable iron, such as ferrous iron (Fe^2+^). In the redox cycle, Fe^2+^ can react with H_2_O_2_ in the Fenton reaction to produce a hydroxyl radical (•OH) that causes oxidative damage [57]. We examined the level of Fe^2+^ in mice in the NAFLD model and found a significant increase in the level of Fe^2+^ compared to control mice, which further aggravated oxidative stress, liver damage, and the development of NAFLD.

Growing evidence from recent studies has revealed an association between iron metabolism and NAFLD. Studies showed that serum ferritin levels were positively associated with NAFLD in an Italian cohort of patients [3] and the Korean general population [27], while the present study showed a nonsignificant negative association between serum ferritin and the risk of NAFLD in the general population of the United States. This difference might be a limitation in that the samples were only from the U.S. population collected by the NHANES 2017–2018. In addition, ferritin has been reported to be an acute-phase reactant protein [66, 67] whose expression increases during inflammation and may be limited in the assessment of iron stores. Although sTfR is less affected by inflammation, there is a lack of accuracy and little broad consensus regarding the use of sTfR in evaluating iron status [68]. TSAT, which is less affected by inflammation [69], is a more useful biomarker in assessing total body iron status [70] and has a certain value in providing prognosis and prediction information. It has been reported that low TSAT levels are associated with an increased risk of BMI [23] and increased mortality among patients with chronic heart failure (CHF) who have normal serum iron levels [71]. In addition, Campodonico et al. suggested that a TSAT level < 20% identifies heart failure patients with the poorest survival rate [72]. Kovesdy et al. found that decreased TSAT correlated with ID has become the most common nutritional disorder, frequently occurring in chronic inflammatory diseases and affecting a subset of patients with CHF, chronic kidney disease (CKD), and inflammatory bowel disease (IBD) [73]. NAFLD is accompanied by chronic inflammation when it progresses to the more severe form of NASH [7]. In our study, decreased TSAT levels were significantly associated with a high risk of NAFLD.

A significant association between TSAT and NAFLD was found among participants who aged < 60 years, female, Mexican American, without hypertension, without DM, and who had a BMI ≥ 30 kg/m^2^ (*P* for trend < 0.05) shown by subgroup analysis. It should be noted that statistical significance of the association in the Mexican American, possibly due to the overall younger age of participants in the Mexican population as compared with other races. Additionally, the association of TSAT and NAFLD differed by age, sex, self-reported race, and BMI (*P* for interaction < 0.05).

In an animal NAFLD model [11], rats fed a high-fructose diet showed decreased serum levels of TSAT, SI, and SF, showing systemic iron deficiency. The authors also observed iron dysregulation in the livers of these mice [11]. The liver is essential for iron sensing, transport, and regulation and orchestrates systemic iron balance by secreting hepcidin [18, 74]. In the circumstance of decreased iron status, the liver can sense this change and reduce the expression of hepcidin, thereby increasing iron bioavailability [17, 75]. It was reported that a reduced liver hepcidin mRNA level was associated with low TSAT levels [60]. Consistently, in our study, hepatic hepcidin mRNA levels were decreased in mice and patients with NAFLD and correlated with decreased serum TSAT levels in mice. In NAFLD, possibly due to a decrease in TSAT, the liver reduces hepcidin mRNA expression in response to this change, reducing ferroportin degradation, allowing more iron to be transported into the blood, and increasing iron bioavailability [17, 75]. Therefore, we concluded that decreased serum TSAT levels and reduced hepatic hepcidin expression indicate decreased systemic iron status and hepatic iron dysregulation in NAFLD, which might provide additional information on biomarkers for the diagnosis of NAFLD.

The current cross-sectional study enrolled a large number of adult participants, comprehensively analyzed the relationship between iron metabolism indicators and NAFLD and showed a significant negative correlation between serum TSAT and NAFLD, which may provide some information for the diagnosis of NAFLD and guidance for appropriate iron supplementation for patients with NAFLD. Nevertheless, there still exist some limitations that cannot be ignored. First, NAFLD status was defined using the U.S. fatty liver index rather than biopsy, which might contribute to inclusion bias among NAFLD patients. Second, a cross-sectional study design does not allow the establishment of causality. Thus, longitudinal studies are needed to determine whether low TSAT levels could be a good biomarker of NAFLD or if it is just a consequence of liver function alteration. Additionally, unmeasured confounders associated with NAFLD might have affected the results despite adjusting for covariates in the regression models. In the future, the impact of iron metabolism on NAFLD and its specific manifestations in the general population requires further prospective studies.

## Conclusion

Decreased serum TSAT levels and hepatic hepcidin expression were observed in both patients and mice with NAFLD. Decreased serum TSAT levels are associated with a higher risk of NAFLD in the U.S. general population. The association was statistically significant among participants who were younger than 60 years, woman, and Mexican American, and those who had a BMI ≥ 30 kg/m^2^.

## Supplementary Information

**Additional file 1: Figure S1** The correlation heatmap of biomarkers of iron metabolism and NAFLD.

**Additional file 2: Figure S2** LASSO regression model screening potential predictors of NAFLD. **a** LASSO regression model cross-validation plot. Draw a vertical line at the optimum with the minimum criterion and 1se of the minimum criterion. When λ = 0.020, we get 13 variables, including TATS, for further analysis. **b** Coefficient profile plot of predictors. Finally, 13 variables including TSAT, were selected at the optimal lambda. LASSO, least absolute shrinkage and selection operator.

**Additional file 3: Figure S3** Restricted cubic spline plots of the association between the TATS and the risk of NAFLD. **a** Analyses were adjusted for age, BMI, waist, Alt, Ast, HDL-C, HbA1c, protein intake, sex, education level, DM, and alcohol user. **b** Analyses were adjusted for age, BUN, smoker, waist, Alt, HbA1C, sex, TC, alcohol user, HDL-C, education level, and dietary Vitamin C intake. Solid and dashed lines represent the log-transformed odds ratios and the corresponding 95% confidence intervals. The level of TSAT was log10 transformed.

**Additional file 4: Figure S4** The establishment of the NAFLD model in mice. **a–c** Mice were fed a normal chow or high-fat-fructose diet for 30 weeks (n = 5 for each group). **a** Body weight of mice after 30 weeks of feeding. **b** Levels of hepatic TG (Left) and TC (Right). **c** Levels of hepatic MDA.

**Additional file 5: Table S1** The characteristics of the populations between the those with missing values and those without.

**Additional file 6: Table S2** Multivariable logistic regression of the associations between Iron metabolism and NAFLD.

**Additional file 7: Table S3** Multivariable stepwise regression analysis for the association between TSAT and NAFLD.

## Data Availability

The data used in this study are available upon reasonable request to the corresponding author, as well as in the NHANES (https://www.cdc.gov/nchs/nhanes/).

## Abbreviations

NAFLD: Non-alcoholic fatty liver disease
NASH: Non-alcoholic steatohepatitis
NHANES: National Health and Nutrition Examination Surveys
FLI: Fatty Liver Index
BMI: Body Mass Index
SI: Serum iron
TSAT: Transferrin saturation
sTfR: Soluble transferrin receptor
SF: Serum ferritin
Family PIR: Family poverty income ratio
DM: Diabetes mellitus
PA: Physical activity
Hb: Hemoglobin
hs -CRP: High-sensitivity C-reactive protein
HbA1c: Glycosylated hemoglobin
ALT: Alanine aminotransferase
AST: Aspartate aminotransferase
GGT: Gamma-glutamyl transpeptidase
TC: Total cholesterol
TG: Triglycerides
HDL-C: High density lipoprotein-cholesterol
BUN: Blood urea nitrogen
UA: Uric acid
Scr: Serum creatinine
eGFR: Estimated glomerular filtration rate
uACR: Urinary albumin creatinine ratio
MDA: Malonic dialdehyde
