# Supplementary figures and images for: Association between iron metabolism and non-alcoholic fatty liver disease: results from the National Health and Nutrition Examination Survey (NHANES 2017–2018) and a controlled animal study
Source: Nutr Metab (Lond). 2022 Dec 13;19:81. doi: 10.1186/s12986-022-00715-y (PMC9749311; doi:10.1186/s12986-022-00715-y)

## Slide 1
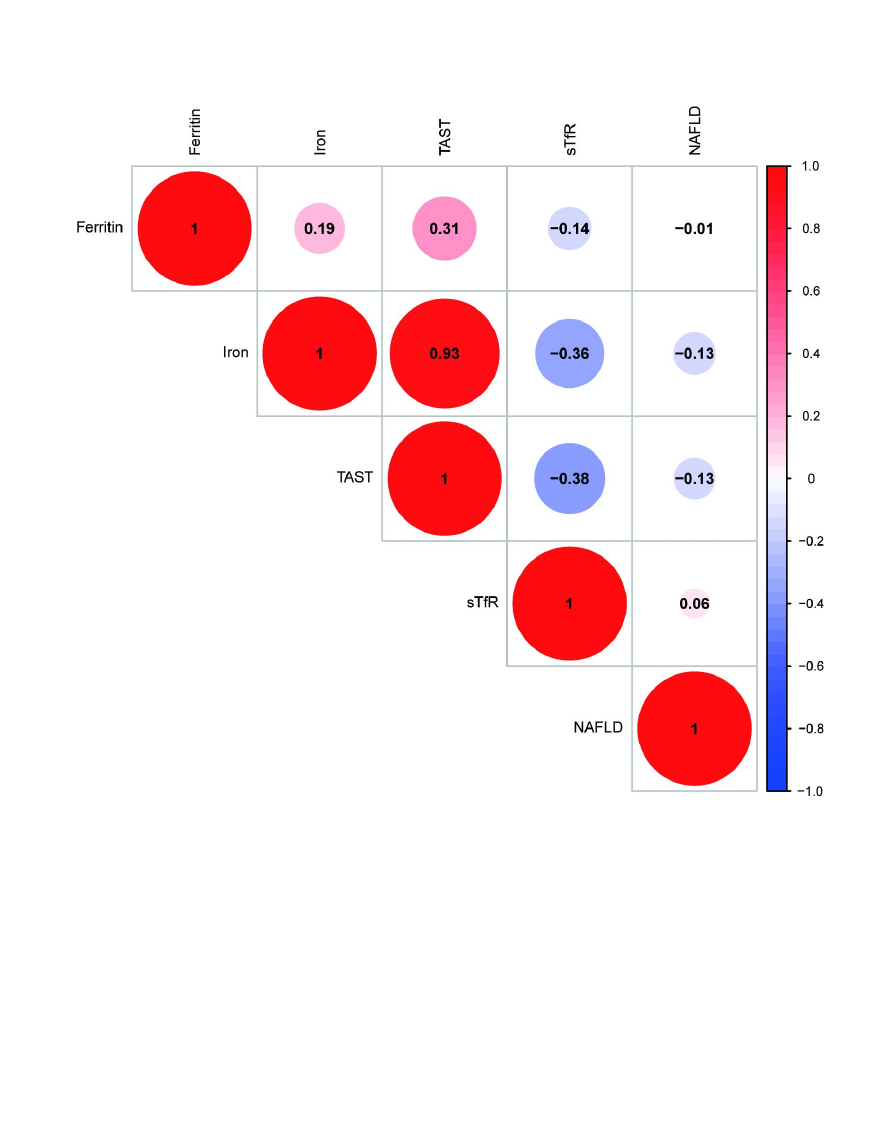

Supplement: Supplementary file 1 — Additional file 1: Figure S1 The correlation heatmap of biomarkers of iron metabolism and NAFLD. [file 12986_2022_715_MOESM1_ESM.pptx]

## Slide 1
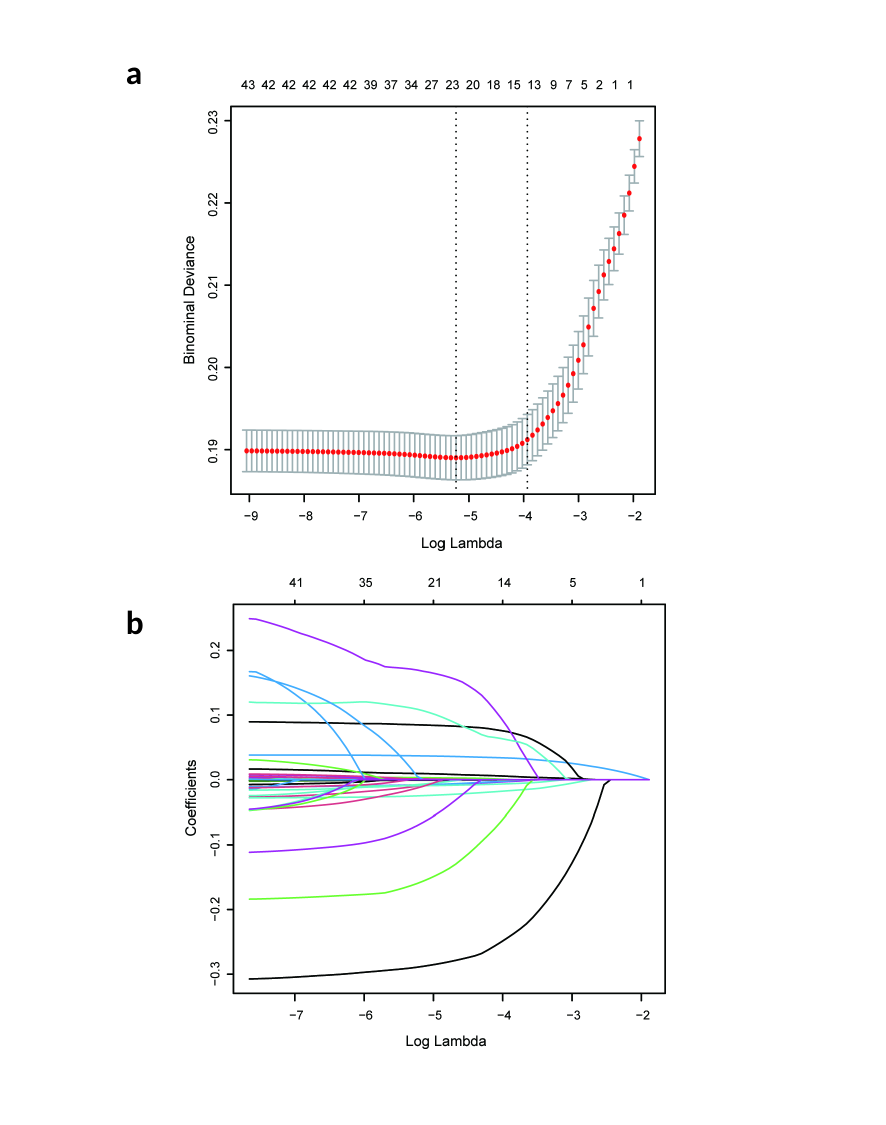

a
b

Supplement: Supplementary file 2 — Additional file 2: Figure S2 LASSO regression model screening potential predictors of NAFLD. a LASSO regression model cross-validation plot. Draw a vertical line at the optimum with the minimum criterion and 1se of the minimum criterion. When λ = 0.020, we get 13 variables, including TATS, for further analysis. b Coefficient profile plot of predictors. Finally, 13 variables including TSAT, were selected at the optimal lambda. LASSO, least absolute shrinkage and selection operator. [file 12986_2022_715_MOESM2_ESM.pptx]

## Slide 1
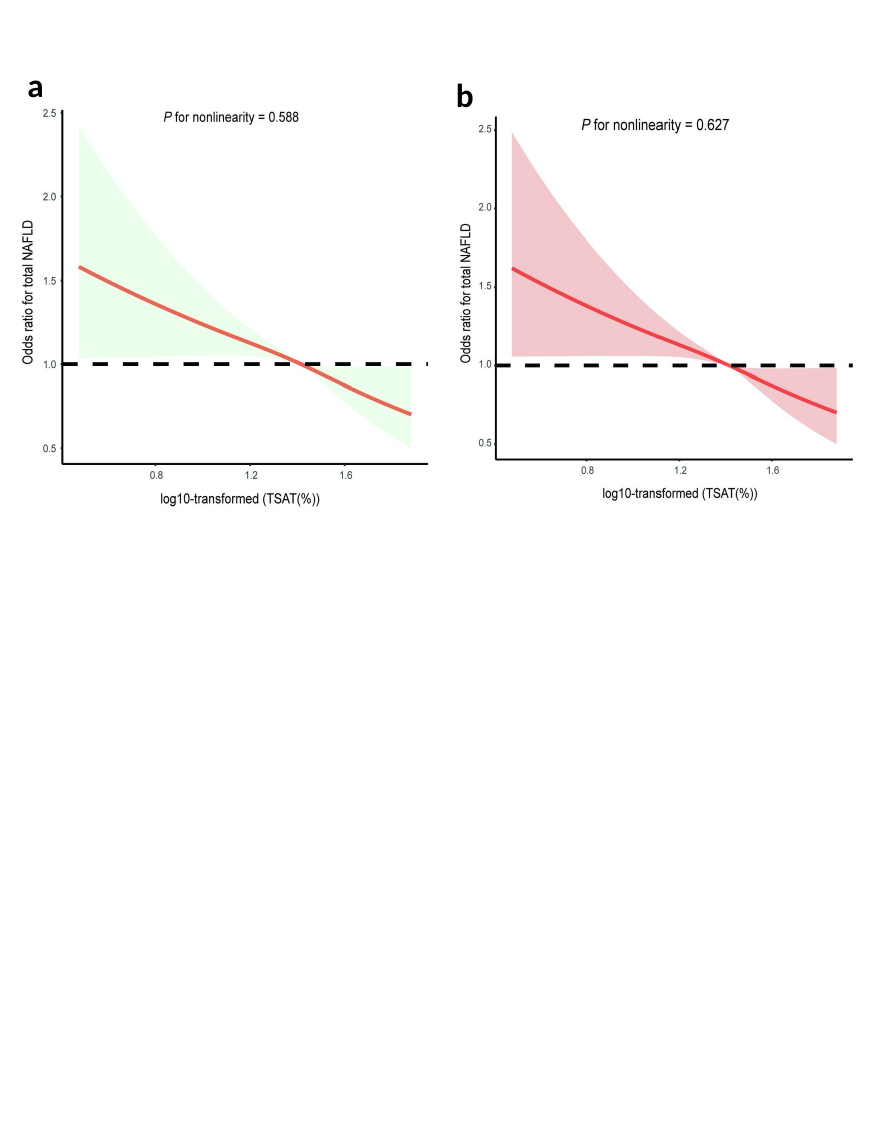

a
b

Supplement: Supplementary file 3 — Additional file 3: Figure S3 Restricted cubic spline plots of the association between the TATS and the risk of NAFLD. a Analyses were adjusted for age, BMI, waist, Alt, Ast, HDL-C, HbA1c, protein intake, sex, education level, DM, and alcohol user. b Analyses were adjusted for age, BUN, smoker, waist, Alt, HbA1C, sex, TC, alcohol user, HDL-C, education level, and dietary Vitamin C intake. Solid and dashed lines represent the log-transformed odds ratios and the corresponding 95% confidence intervals. The level of TSAT was log10 transformed. [file 12986_2022_715_MOESM3_ESM.pptx]

## Slide 1
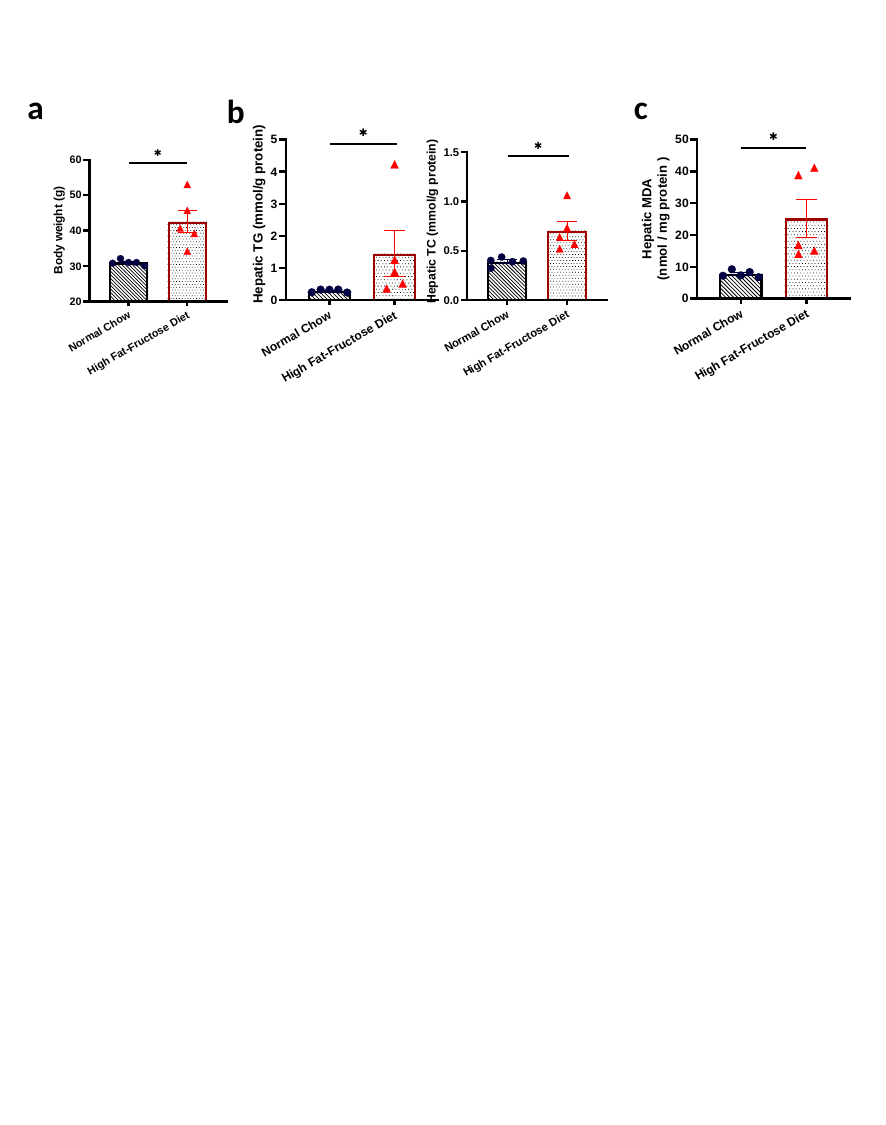

a
c
b

Supplement: Supplementary file 4 — Additional file 4: Figure S4 The establishment of the NAFLD model in mice. a–c Mice were fed a normal chow or high-fat-fructose diet for 30 weeks (n = 5 for each group). a Body weight of mice after 30 weeks of feeding. b Levels of hepatic TG (Left) and TC (Right). c Levels of hepatic MDA. [file 12986_2022_715_MOESM4_ESM.pptx]
