# Supplementary material for: Association between iron metabolism and non-alcoholic fatty liver disease: results from the National Health and Nutrition Examination Survey (NHANES 2017–2018) and a controlled animal study
Source: Nutr Metab (Lond). 2022 Dec 13;19:81. doi: 10.1186/s12986-022-00715-y (PMC9749311; doi:10.1186/s12986-022-00715-y)
Supplement: Supplementary file 5 — Additional file 5: Table S1 The characteristics of the populations between the those with missing values and those without. [file 12986_2022_715_MOESM5_ESM.docx]

| **Supplementary Table 1.** The characteristics of the populations between the those with missing values and those without. | | | | |
| --- | --- | --- | --- | --- |
| Variable | Individuals with  missing values (n=918) | Individuals without  missing values (n=4853) | *P*-value |  |
| Age, years | 44.32 ± 22.98 | 44.43 ± 0.62 | 0.062 |  |
| Sex, % |  |  |  |  |
| Male | 423 (46.1%) | 2705 (49.3%) | 0.068 |  |
| Female | 495 (53.9%) | 2778 (50.7%) |  |  |
| Race, % |  |  |  |  |
| Mexican American | 115 (12.5%) | 810 (14.8%) | 0.147 |  |
| Other Hispanic | 88 (9.6%) | 511 (9.3%) |  |  |
| Non-Hispanic Black | 231 (25.2%) | 1216 (22.2%) |  |  |
| Non-Hispanic White | 297 (32.4%) | 1868 (34.1%) |  |  |
| Other Race | 187 (20.4%) | 1078 (19.7%) |  |  |
| Family PIR | 3.02 ± 0.16 | 3.00 ± 0.06 | 0.083 |  |
| Education level, % |  |  | 0.066 |  |
| less than high school | 308 (33.6%) | 1632 (29.8%) |  |  |
| High school | 198 (21.6%) | 1212 (22.1%) |  |  |
| more than high school | 412 (44.9%) | 2639 (48.1%) |  |  |
| Marital status, % |  |  | 0.309 |  |
| Having a partner | 499 (54.4%) | 3031 (55.3%) |  |  |
| No partner | 196 (21.4%) | 1054 (19.2%) |  |  |
| Unmarried | 223 (24.2%) | 1398 (25.5%) |  |  |
| Hypertension, % |  |  |  |  |
| No | 560 (8.7%) | 3342 (61.0%) | 0.977 |  |
| Yes | 358 (5.6%) | 2141 (39.0%) |  |  |
| DM, % |  |  | 0.573 |  |
| No | 759 (11.9%) | 4491 (81.9%) |  |  |
| Yes | 159 (2.5%) | 992 (12.8%) |  |  |
| Smoker, % |  |  | 0.096 |  |
| No | 580 (63.2%) | 3328 (60.7%) |  |  |
| Former | 165 (18.0%) | 1157 (21.1%) |  |  |
| Now | 173 (18.8%) | 998 (18.2%) |  |  |
| Alcohol user, % |  |  | 0.012 |  |
| No | 199 (21.7%) | 873 (15.9%) |  |  |
| Mild | 363 (39.5%) | 2233 (40.7%) |  |  |
| Moderate | 179 (19.5%) | 1045 (19.1%) |  |  |
| Heavy | 177 (19.3%) | 1332 (24.3%) |  |  |
| CHD, % |  |  | 0.025 |  |
| No | 872 (95.0%) | 5291 (96.5%) |  |  |
| Yes | 46 (5.0%) | 192 (3.5%) |  |  |
| CHF, % |  |  | 0.056 |  |
| No | 886 (96.5%) | 5360 (97.8%) |  |  |
| Yes | 32 (3.5%) | 123 (2.2%) |  |  |
| Angina, % |  |  | 0.178 |  |
| No | 874 (96.8%) | 5356 (97.7%) |  |  |
| Yes | 29 (3.2%) | 127 (2.3%) |  |  |
| Heart attack, % |  |  | 0.057 |  |
| No | 870 (94.8%) | 5278 (96.3%) |  |  |
| Yes | 48 (5.2%) | 205 (3.7%) |  |  |
| Stroke, % |  |  | 0.071 |  |
| No | 873 (95.1%) | 5274 (96.2%) |  |  |
| Yes | 45 (4.9%) | 209 (3.8%) |  |  |
| PA, % |  |  | 0.320 |  |
| No | 504 (54.9%) | 2855 (52.1%) |  |  |
| Moderate | 193 (21.0%) | 1211 (22.1%) |  |  |
| Both | 175 (19.1%) | 1164 (21.2%) |  |  |
| Vigorous | 46 (5.0%) | 253 (4.6%) |  |  |
| Mean energy | 2085.09 ± 20.09 | 2074.41 ± 21.10 | 0.952 |  |
| intake (kcal/day) |  |  |  |  |
| Protein intake, g | 78.09 ± 32.50 | 79.99 ± 1.15 | 0.137 |  |
| Folic acid intake, mcg | 176.44 ± 148.03 | 175.01 ± 3.76 | 0.554 |  |
| Vitamin B12 intake, mcg | 4.46 ± 3.58 | 4.76 ± 0.08 | 0.165 |  |
| Vitamin C intake, mg | 74.56 ± 70.70 | 73.63 ± 1.37 | 0.771 |  |
| Iron intake, mg | 13.78 ± 7.15 | 14.12 ± 0.20 | 0.288 |  |
| BMI, kg/m^2^ | 28.84 ± 8.14 | 29.14 ± 0.24 | 0.079 |  |
| Waist circumference, cm | 97.69 ± 19.58 | 98.78 ± 0.64 | 0.004 |  |
| Hb, g/dL | 13.65 ± 1.62 | 14.21 ± 0.06 | 0.096 |  |
| Hs CRP, mg/L | 4.94 ± 9.27 | 3.59 ± 0.15 | 0.001 |  |
| HbA1c, % | 5.72 ± 0.99 | 5.63 ± 0.02 | 0.476 |  |
| ALT, U/L | 21.20 ± 13.62 | 22.52 ± 0.38 | 0.421 |  |
| AST, U/L | 21.01 ± 13.373 | 22.09 ± 0.26 | 0.546 |  |
| GGT, U/L | 29.26 ± 38.15 | 28.26 ± 0.50 | 0.341 |  |
| TC, mg/dL | 183.89 ± 46.975 | 185.50 ± 1.59 | 0.169 |  |
| TG, mg/dL | 135.99 ± 145.85 | 136.93 ± 3.18 | 0.423 |  |
| HDL-C, mmol/L | 1.31 ± 0.02 | 1.38 ± 0.01 | 0.004 |  |
| BUN, mg/dL | 14.39 ± 6.99 | 14.59 ± 0.17 | 0.067 |  |
| UA, mg/dL | 5.45 ± 1.62 | 5.34 ± 0.04 | 0.423 |  |
| Scr, mg/dL | 0.86 ± 0.03 | 0.86 ± 0.01 | 0.528 |  |
| eGFR, ml/min/1.73m^2^ | 104.58 ± 34.05 | 98.81 ± 0.85 | 0.496 |  |
| uACR | 53.43 ± 288.06 | 34.54 ± 3.26 | 0.844 |  |

Abbreviation: NAFLD, non-alcoholic fatty liver; family PIR, family poverty income ratio; DM, diabetes mellitus; BMI, body mass index, PA, physical activity; Hb, hemoglobin; hs CRP, high-sensitivity C-reactive protein; HbA1c, glycosylated hemoglobin; ALT, alanine aminotransferase; AST, aspartate aminotransferase; GGT, gamma-glutamyl transpeptidase; TC, total cholesterol; TG, triglycerides; HDL-C, high-density lipoprotein-cholesterol, BUN, blood urea nitrogen; UA, uric acid; Scr, serum creatinine; eGFR, estimated glomerular filtration rate; uACR, urinary albumin creatinine ratio.
