# Supplementary material for: Association between iron metabolism and non-alcoholic fatty liver disease: results from the National Health and Nutrition Examination Survey (NHANES 2017–2018) and a controlled animal study
Source: Nutr Metab (Lond). 2022 Dec 13;19:81. doi: 10.1186/s12986-022-00715-y (PMC9749311; doi:10.1186/s12986-022-00715-y)
Supplement: Supplementary file 6 — Additional file 6: Table S2 Multivariable logistic regression of the associations between Iron metabolism and NAFLD. [file 12986_2022_715_MOESM6_ESM.docx]

| **Supplementary Table 2.** Multivariable logistic regression of the associations between Iron metabolism and NAFLD. | | | | | |  |
| --- | --- | --- | --- | --- | --- | --- |
| Iron metabolism | Model 1 |  | Model 2 |  | Model 3 |  |
|  | OR (95%CI) | *P*-value | OR (95%CI) | *P*-value | OR (95%CI) | *P*-value |
| SI (μg/dL) | 0.922 (0.990, 0.994) | <0.001 | 0.993 (0.991, 0.995) | <0.001 | 0.998 (0.996, 0.999) | 1.0710.047 |
| SF (μg/L) | 0.999 (0.998, 1.000) | 0.002 | 0.999 (0.998, 1.000) | 0.002 | 1.000 (0.999, 1.000) | 0.081 |
| TSAT (%) | 0.974 (0.969, 0.979) | <0.001 | 0.977 (0.972, 0.983) | <0.001 | 0.989 (0.983, 0.996) | 0.001 |
| sTfR (mg/L) | 1.071 (1.040, 1.104) | <0.001 | 1.053 (1.022, 1.085) | 0.001 | 1.022 (0.987, 1.058) | 0.218 |

Abbreviation: SI, serum iron; SF, serum ferritin; TSAT, Transferrin saturation; sTfR, soluble transferrin receptor; NAFLD, non-alcoholic fatty liver; Model 1: age and sex. Model 2: Model 1 variables plus race/ethnicity, family poverty-income ratio, marital status, education level, hypertension, diabetes mellitus, smoker, alcohol user. Model 3 was adjusted for Model 2 variables plus body mass index, waist circumference, physical activity, the complication of CHD, CHF, angina pectoris, heart attack, and stroke, mean energy intake, protein intake, folic acid intake, Vitamin B12 intake, Vitamin C intake, Iron intake, high-sensitivity C-reactive protein, glycosylated hemoglobin, alanine aminotransferase, aspartate aminotransferase, gamma-glutamyl transpeptidase, blood urea nitrogen, uric acid, serum creatinine, estimated glomerular filtration rate, urinary albumin creatinine ratio, hemoglobin, high-density lipoprotein-cholesterol, total cholesterol, triglycerides.
