# Supplementary material for: Association between iron metabolism and non-alcoholic fatty liver disease: results from the National Health and Nutrition Examination Survey (NHANES 2017–2018) and a controlled animal study
Source: Nutr Metab (Lond). 2022 Dec 13;19:81. doi: 10.1186/s12986-022-00715-y (PMC9749311; doi:10.1186/s12986-022-00715-y)
Supplement: Supplementary file 7 — Additional file 7: Table S3 Multivariable stepwise regression analysis for the association between TSAT and NAFLD. [file 12986_2022_715_MOESM7_ESM.docx]

| **Supplementary Table 3.** Multivariable stepwise regression analysis for the association between TSAT and NAFLD. | | |
| --- | --- | --- |
| Variables | OR (95%CI) | *P*-value |
| TSAT | 0.989 (0.983, 0.995) | <0.001 |
| Age | 1.012 (1.008, 1.016) | <0.001 |
| BUN | 0.984 (0.972, 0.995) | 0.006 |
| Smoker | 0.887 (0.811, 0.970) | 0.009 |
| Waist circumference | 1.042 (1.038, 1.047) | <0.001 |
| ALT | 0.966 (0.961, 0.971) | <0.001 |
| HbA1C | 1.139 (1.069, 1.214) | <0.001 |
| Sex | 1.253 (1.088, 1.444) | 0.002 |
| TC | 1.002 (1.001, 1.004) | 0.001 |
| Alcohol user | 0.736 (0.688, 0.787) | <0.001 |
| HDL-C | 0.987 (0.981, 0.991) | <0.001 |
| Education level | 0.806 (0.747, 0.869) | <0.001 |
| Dietary Vitamin C | 0.998 (0.997, 0.999) | 0.009 |

Abbreviation: TSAT, Transferrin saturation; NAFLD, non-alcoholic fatty liver; BUN, blood urea nitrogen; ALT, alanine aminotransferase; HbA1c, glycosylated hemoglobin; TC, total cholesterol; AST, aspartate aminotransferase; HDL-C, high-density lipoprotein-cholesterol.
